# Multidrug-Resistant Bacteria on Healthcare Workers’ Uniforms in Hospitals and Long-Term Care Facilities in Cyprus

**DOI:** 10.3390/antibiotics11010049

**Published:** 2021-12-31

**Authors:** Pavlina Lena, Spyridon A. Karageorgos, Panayiota Loutsiou, Annita Poupazi, Demetris Lamnisos, Panagiotis Papageorgis, Constantinos Tsioutis

**Affiliations:** 1 Mpn Unilab Clinical Laboratory, Nicosia 1066, Cyprus; pavlinalena@cytanet.com.cy (P.L.); loutsiou.panayiota@ucy.ac.cy (P.L.); annitapoupazi@hotmail.com (A.P.); 2 Department of Health Sciences, School of Sciences, European University Cyprus, Nicosia 2404, Cyprus; d.lamnisos@euc.ac.cy; 3 School of Medicine, European University Cyprus, Nicosia 2404, Cyprus; s.karageorgos@external.euc.ac.cy; 4 First Department of Pediatrics, “Aghia Sophia” Children’s Hospital, National and Kapodistrian University of Athens, 11527 Athens, Greece; 5 Department of Life Sciences, School of Sciences, European University Cyprus, Nicosia 2404, Cyprus; p.papageorgis@euc.ac.cy

**Keywords:** clothing, attire, textiles, MDRB, MRSA, VRE, ESBL, healthcare-associated infections

## Abstract

Healthcare workers’ (HCW) clothing has been shown to harbor multidrug-resistant bacteria (MDRB) and may contribute to transmission. The aim of this study was to evaluate presence of MDRB on HCW uniforms in Cyprus. A cross-sectional study was carried out in 9 hospital wards and 7 long-term care facilities (LTCFs) in Nicosia, Cyprus, from April–August 2019. Sampling of HCW uniform pockets was conducted at the end of the first shift. Personal hygiene and other habits were recorded during personal interviews. Among 140 sampled HCW (69 from hospitals, 71 from LTCFs), 37 MDRB were identified, including 16 vancomycin-resistant enterococci (VRE), 15 methicillin-resistant *Staphylococcus aureus* (MRSA), 5 extended spectrum b-lactamase (ESBL)-producing bacteria, and 1 carbapenem-resistant *Acinetobacter baumannii*. Presence of MDRB was higher in LTCFs compared to hospitals (*p* = 0.03). Higher MDRB rates in uniforms were noted in HCWs that worked <1 year (41.7% vs. 21.1%) and in HCWs that opted for home laundering (23.5% vs. 12.5%) or visited the toilet during shifts (38.1% vs. 20.2%). Our findings indicate that HCW uniforms harbor MDRB and relevant interventions may reduce transmission risk. We identified LTCFs as an important area for targeted measures. Additional factors associated with HCW practices, characteristics, and attire laundering practices represent areas for improvement, particularly in LTCFs.

## 1. Introduction

Multidrug-resistant bacteria (MDRB) are defined as bacteria that are non-susceptible to agents in at least more than three antimicrobial categories [1]. These bacteria continue to represent a significant public health problems in healthcare settings, with important implications on patient outcomes [2]. Horizontal transmission of MDRB constitutes an important concern in hospital settings and long-term care facilities (LTCF). The Center for Disease Control and Prevention (CDC) categorizes three primary routes of transmission of microorganisms in healthcare settings: through contact (direct or indirect for example, through textiles), through respiratory droplets, and airborne [3,4].

MDRB, such as methicillin-resistant *Staphylococcus aureus* (MRSA), have been found to survive for long on commonly used hospital fabrics, including scrub suits, lab coats, and hospital privacy drapes. Similarly, studies have demonstrated that HCW attire and devices in hospitals and LTCFs harbor pathogens and may contribute to the transmission cycle [5]. HCW frequently wipe their hands on their uniforms and have contact with patient belongings and linen often without the use of protective barriers, thus unknowingly carrying infectious microorganisms. Since a large proportion of HCW wear their uniforms to and from work, there is potential risk of transporting microorganisms between hospital and community environments [6].

The presence of pathogens including MDRB on HCW attire underscores the need for further investigation and targeted measures to limit their spread [7]. Previous systematic reviews [5,8] showed that HCW attire and devices may harbor bacteria which in turn can act as vectors in the transmission of pathogens in healthcare settings.

Cyprus is considered a high prevalence country for MDRB. In fact, among patients with healthcare-associated infections (HAIs), MRSA accounted for 40.2% of *S. aureus* isolates, vancomycin resistance was detected in 59.1% of *Enterococci*, and *Klebsiella pneumoniae* isolates were 32.2% multidrug-resistant and 21.8% carbapenem-resistant [9]. The reported high rates of MDRB in Cyprus raise serious concerns for different modes of transmission of MDRB within healthcare settings and warrant further studies to identify areas for intervention.

Βased on the above, our study aimed to record evidence regarding the presence of MDRB on HCW attire in healthcare settings in Cyprus and to identify areas for improvement.

## 2. Results

### 2.1. Participants

A total of 140 participants were included in the study; 69 (49.3%) were from hospitals and 71 (50.7%) were from LTCFs. The great majority (*n* = 131, 93.6%) were nurses (university graduates), 5 (3.6%) were cleaning staff, and 4 (2.8%) were care assistants. No doctors were included among study participants, as at the time of sampling, no doctors were present, or if present, they were not wearing scrubs or white coats. Most (63 (45%)) had working experience for 1–5 years, 34 (24.3%) worked from 6–10 years, and 31 (22.1%) had been working for more than 10 years. Only 12 (8.6%) had been working for less than a year. Most HCW (77.1%) were females.

### 2.2. Laundering and Hygiene Practices

Most participants (*n* = 111, 79.3%) reported changing their uniforms daily, 24 (17%) every 2 days, and 5 (3.6%) every more than 2 days. The vast majority (*n* = 132, 94.3%) reported washing their uniforms at home and only 8 (5.7%) used the work laundry facilities. Concerning staff movement during their shift, 112 (80%) visited multiple sites, 21 (15%) visited the toilet during the shift, 6 (4.3%) visited other wards, and 1 (0.7%) visited the cafeteria. Regarding personal hygiene, 137 (97.9%) reported washing their hands more than 5 times during their shift and only 3 (2.1%) reported washing their hands 1–5 times (Table 1).

A higher percentage of HCW working for less than a year was recorded in LTCF (15.5% vs. 1.4%) and a higher percentage of HCW with more than 10 years of experience was recorded in hospitals (31.9% vs. 12.7%, *p* < 0.01). The vast majority of HCW (94.3%) opted for home laundering (100% for LTCF vs. 88.4% for hospital, *p* < 0.01), as opposed to the rest who used the hospital facilities, and 80% of participants visited multiple sites (87.3% for LTCF vs. 72.5% for hospitals, *p* < 0.03), while 15% of HCW visited the toilet during their shift (12.7% for LTCF vs. 17.4% for hospital).

### 2.3. Isolates

Among a total of 280 samples from 140 HCW, 37 MDRB (13.2%) were identified in 32 HCW (Table 2). Isolation of the 37 MDRB were as follows: 16 (43.2%) were VRE, 15 (40.5%) were MRSA, 5 (13.5%) were ESBL producers, and 1 (2.7%) was carbapenem-resistant *Acinetobacter baumannii* (CRAB).

Out of the 16 VRE isolates, 14 were *Enterococcus faecium* and the remaining 2 were *Enterococcus faecalis.* Detection of phenotypes against the glycopeptide antibiotic family presented with 15 VanA-like and 1 VanB-like. All *Enterococcus* isolates were susceptible to linezolid and tigecycline. The ESBL-producing organisms were *Klebsiella pneumoniae* (*n* = 3), *Leclercia adecarboxylata* (*n* = 3), *Enterobacter cloacae* ssp. *dissolvens* (*n* = 1). In 4 HCW, more than one isolate was identified (Table 2).

Presence of MDRB was higher in LTCFs (*n* = 27) compared to hospitals (*n* = 10) (*p* = 0.03). MRSA isolation was not different between hospitals and LTCF (*p* = 0.61), but VRE- and ESBL-producing organism isolation were higher in LTCF (*p* = 0.01 and *p* = 0.06 respectively). Higher prevalence of isolated organisms from uniforms was noted in HCWs that had worked less than a year (41.7% vs. 21.1%), in HCWs that opted for home laundering (23.5% vs. 12.5%), and among HCWs that visited the toilet during their shifts (38.1% vs. 20.2%) (Table 3).

## 3. Discussion

Our study showed that a significant proportion of HCW uniforms may harbor MDRB and represent a potential transmission vehicle. Three out of 16 (4.3%) HCW from hospitals had VRE on their pockets as opposed to 13 out of 16 (18.3%) from LTCF. MRSA isolation rates were similar between hospitals and LTCF (10.1% and 14.1%, respectively), whereas ESBL-producing bacteria were exclusively isolated in HCW uniforms from LTCF. It is also worth mentioning that in the 2 intensive care units included in our study, the only isolated MDRB were MRSA.

MRSA has been reported on HCW attire in several other studies, ranging between 1.3–79% on scrubs and 0–19.1% on uniforms [8]. On the other hand, our results point towards notably higher rates of VRE- and ESBL-producing bacteria in LTCF compared to hospitals, suggesting that these settings may be a reservoir of resistant bacteria. These differences may be attributed to various reasons: Firstly, in Cyprus, several LTCFs function as nursing homes for the elderly, leading to a high patient mix. Secondly, LTCFs have a higher proportion of care assistants, who may lack training in appropriate infection control and laundering protocols. Finally, hospitals as opposed to LTCF have infection control teams, established infection control protocols and frequent training of HCW on infection control practices, especially in high-risk units. 

VRE represented nearly half of all isolated MDRB in our study. The presence of high numbers of VRE in LTCF is reason for concern, taking into consideration the high rates of invasive VRE isolates in hospitals in Cyprus and the intrinsic resistant patterns of enterococci to several antimicrobial classes [10,11]. As reported in the latest surveillance report of antimicrobial resistance in Europe in 2018, Cyprus portrays an alarming upward trend in vancomycin resistance among nosocomial *E. faecium* isolates, with reported resistance rates from 28.6% in 2015 to 59.1% in 2018, ranking first in the EU/EEA [9]. In comparison, the EU/EEA mean percentage of VRE is 18.3%. The WHO has listed vancomycin-resistant *E. faecium* as a high priority pathogen which constitutes a major challenge for infection control [12]. 

Since enterococci are part of the microbial flora, they can be easily disseminated in healthcare settings especially in LTCFs, where the residents are often bedbound and require constant care. In a study by Jackson et al. [11], the incidence of VRE isolated on HCW gloves or gowns was 15%, noting that direct patient contact was the major cause of contamination. Similarly, we isolated VRE in 11.4% of the HCW uniforms sampled in LTCFs. Although VRE colonization rates remain unknown in our settings, it is reasonable to conclude a high proportion of colonization among patients, particularly in LTCFs, thus serving as reservoirs in maintaining the cycle of transmission. Furthermore, as VRE are capable of survival in the environment and can transfer their resistance via plasmids to MRSA strains, prompt early detection and enhanced efforts to decrease colonization are critical [13].

According to a recent review [14], the median worldwide prevalence of colonization of LTCF residents with ESBL-producing Enterobacterales was 11.6% (interquartile range, 5.5–24.5%), with significant geographic variation between continents. In our study, 7% of HCW in LTCFs harbored ESBL-producing organisms on their uniforms, whereas none were isolated in hospitals, implying different colonization rates of patients and fomites between hospitals and LTCFs. This is in line with previous studies reporting much higher ESBL colonization rates of LTCF residents compared to hospitals [15,16]. 

In Cyprus, national recommendations on MDRB focus on strategies for prevention of spread in healthcare settings [17], whereas preadmission screening is based on local hospital practices and is limited to MRSA screening of patients transferred from hospitals to LTCFs. This might account for the similar rates of MRSA isolation on HCW attire in hospitals and LTCFs. However, our findings imply that there is need to consider routine preadmission screening for MDRB, as well as to study HCW attire laundering practices during routine health control monitoring. To this end, especially in high prevalence areas such as Cyprus, it is important to establish national practices for HCW attire laundering in addition to recommendations for the prevention and control of MDRB in healthcare settings. Strict protocols on infection control practices and training are needed in LTCFs and must be monitored by the local health authorities. These protocols must be adhered to international recommendations and can be adapted to national or regional characteristics [4]. This is further supported by the observed characteristics and practices of HCW in our study, including home laundering practices, increased mobility during shifts and the fact that most MDRB were isolated in younger HCWs. 

Established HCW uniform washing protocols [18] accept both in-house and home laundering practices. These practices must be part of the staff training with clear washing instructions, such as temperature, addition of chemicals. However, a point of further concern is the fact that HCW wear their uniforms to and from work. The spread of infectious particles from the uniforms to the environment when leaving work cannot be dismissed, as well as the acquisition of microorganisms from the environment coming to work. A previous study recovered multidrug-resistant staphylococci from frequently touched surfaces in public areas of community areas and hospitals in East and West London [19]. This is an indication of the risks of acquiring resistant organisms on uniforms when moving to and from work using public transport, or simply by touching frequently touched surfaces.

Our results should be interpreted considering certain limitations. Sampling took place during summer months where temperatures are high; therefore, seasonal variations were not taken into consideration. Most participants stated using home laundering and thus, sampling at the beginning of their shifts and additional details of laundering practices (temperature, type of detergent, use of bleach, tumble drying) could have contributed to evaluate the effectiveness of in-house laundering. Low numbers in HCW, as well as the fact that our results reflect local epidemiology and practices, may have affected representativeness, generalizability, and further statistical analyses. Since the survey was conducted anonymously, repeat testing was not possible. Our study design did not allow for processing of MRSA isolates to distinguish between hospital- and community-acquired strains. Finally, phenotypic characterisation of MDRB isolates precluded clonal analysis. On the other hand, our study includes data from multisite, diverse healthcare settings from hospital wards, ICU, and LTCF in the largest city of the country, following a sound methodology and a standardized sampling technique. Further studies should assess the transmission potential between colonized attire and patients.

Conclusively, the current study confirmed the presence of MDRB on HCW uniforms in healthcare settings. Our data revealed that LTCFs are an important reservoir of MDRB, providing specific targets for intervention and raising the need for enhancement of infection control and laundering policies. Further studies are warranted to increase understanding of the reservoirs and modes of transmission, as well as the contribution of attire type and laundering protocols in MDRB colonization and transmission.

## 4. Materials and Methods

### 4.1. Study Aims

The aim of the current study is to determine the presence of MDRB on HCW attire and to evaluate the relationship between HCW practices and MDRB presence.

### 4.2. Setting

We conducted a cross-sectional study between April–August 2019 in 2 tertiary care hospitals (one public, one private) and 7 LTCF in Nicosia, Cyprus. Hospital wards included in the study were: 4 internal medicine wards, 2 orthopedic wards, 2 intensive care units (ICU; one general ICU and one cardiac ICU), 1 burn unit, and 7 LTCF. Time of the sampling was at the end of the first morning shift (11:00–13:00).

### 4.3. Participants

Participating HCW were permanent members of staff, present at their posts at the time of sampling, who consented to sampling and to anonymously complete the questionnaire. Following consent, participants completed a questionnaire which recorded data related to demographic details and daily practices: sex, place of residence, dominant hand, hospital and ward or LTCF, frequency of uniform washing, use of white coats or scrubs, home or in-house laundering, frequency of hand washing during shift, and visits to other locations during shift (toilet, cafeteria, other wards). Selection of HCW was random homogenous convenient, cross-sectional, and anonymous, on a voluntary basis.

### 4.4. Pilot Study

Due to the unknown prevalence of MDRB on the uniforms of HCWs, a pilot study consisting of 20 samples was performed using both methods of processing samples (enrichment vs. non enrichment of the swabs in brain–heart infusion broth, BHI, prior to incubation). These 20 samples yielded MRSA in 16.6% of samples cultured via the enrichment process and none from the direct inoculation of the swabs on the selective media.

The estimation of sample size was calculated using Cochran’s formula [20]. Considering probabilities of 10%, 20%, and 30% for prevalence of MDRB on HCW uniforms, then, for 10%, 138 samples were necessary; for 20%, 244 samples were necessary; and for 30%, 322 samples were necessary. Therefore, the minimum accepted sample size was set at minimum of 138 samples. 

### 4.5. Sampling Procedure and Microbiology

Sterile wooden tampon swabs were pre-moistened in 1 ml of maximum recovery diluent (MRD). Two swabs were taken from each participant from 2 points (left and right pocket) of their top uniform. Each swab was used by passing in a rotating manner 3 times back and forth on the top part of the pocket (20 × 5 cm) and repeated, returning vertically on each of the 2 pockets of the suit. (ISO 14698-1:2003)

Each swab was placed in 1 mL of MRD, sealed, and numbered. The samples were kept in cooler bags until their transportation to mpn UNILAB Ltd. clinical laboratory within one hour. Upon arrival to the laboratory, the swabs were topped up with BHI for overnight enrichment at 37 °C. Following enrichment, the swabs were streaked on specialized chromogenic media: Liofilhem: CHROMOagar, MRSA medium, ChromoVRE, ChromoESBL, and CLED BEVIS. Inoculated agar plates were further incubated at 37 °C for 24–48 h. All colonies were sub-cultured onto Columbia agar containing 5% horse blood and processed for identification using the VITEK 2 SYSTEM. GP gram-positive-ID and GN gram negative-ID cards were used together with their antibiotic sensitivity cards AST N215, and AST P580.

### 4.6. Antimicrobial Susceptibility Testing

The automated VITEK 2 system for the rapid identification and antimicrobial susceptibility system using the advanced expert system (AES) detects any inconsistencies between the isolates and their sensitivities, ascertaining the antimicrobial phenotypes [21]. Interpretation of the susceptibility testing aims to analyze the patterns, rather than the individual results for each antibiotic in order to predict any underlying resistance, allowing any anomalies between the phenotype and the organism to be considered [22].

For *Staphylococcus aureus*, the following antimicrobials were tested: benzyl penicillin, oxacillin, gentamicin, tobramycin, levofloxacin, moxifloxacin, erythromycin, clindamycin, linezolid, teicoplanin, tigecycline, fosfomycin, fusidic acid, rifampicin, and trimethoprim-sulfamethoxazole (TMP-SMX). 

The MRSA flag was based on the phenotypic analysis against the beta-lactam antibiotic family with the detected phenotype of the modification of PBP (mecA) and the positive cefoxitin screen. For enterococci, the antimicrobials tested were azithromycin, clarithromycin, linezolid, teicoplanin, vancomycin, and tigecycline. Resistance to vancomycin was flagged as the VRE strain. Enterobacteriaceae tested and flagged as extended spectrum lactamase-producing (ESBL) organisms were tested for: ampicillin, amoxicillin/clavulanic acid, piperacillin/tazobactam, cefuroxime, cefuroxime axetil, cefpodoxime, cefotaxime, ceftazidime, imipenem, meropenem, gentamicin, tobramycin, ciprofloxacin, norfloxacin, and TMP-SMX.

The MIC interpretation guidelines by the VITEK 2 system Version 08.01 (bioMérieux) in accordance with the EUCAST standards [23].

### 4.7. Definitions

For the purpose of this study, “uniform” was defined as clothing worn by HCW for everyday practice in clinical environments and included scrub suits, white coats, or jackets. Left and right pockets were a prerequisite for the inclusion of uniforms in the study.

### 4.8. Ethics

Approval from the Bioethical committee of Cyprus was obtained (decision number “EEBK EΠ 2018.01.155”). Individual agreement for participation of the study was also obtained from each facility management. Participants accepted to take part voluntarily, signed a consent form before sampling and before completing the questionnaire (Supplementary Materials). All recorded information was anonymized and coded and kept in a password-protected server.

### 4.9. Statistical Analysis

Differences in participants’ characteristics and practices across the health care settings were evaluated with the Pearson’s chi-squared test and Fisher’s exact test. The same statistical tests were used to evaluate differences in the prevalence of MDRB across participant’s characteristics and practices. If the same isolates were retrieved on both pockets from the same HCW, these were merged as one sample. Statistical analysis was performed with the SPSS statistical package, version 26 (IBM Corp., Armonk, NY, USA).

## Figures and Tables

**Table 1:** Participants’ characteristics and practices.

Characteristics/Practices	Healthcare Setting			
	Total (*n* = 140)	Hospital (*n* = 69)	LTCF (*n* = 71)	
	*n* (%)	*n* (%)	*n* (%)	*p*-Value
Type of HCW				0.05
Nurses	131 (93.6%)	68 (98.6)	63 (88.7)	
Cleaning staff	5 (3.6%)	1 (1.4)	4 (5.6)	
Care assistants	4 (2.9%)	0 (0.0)	4 (5.6)	
Years of work				<0.01
<1	12 (8.6%)	1 (1.4%)	11 (15.5%)	
1–5	63 (45%)	29 (42.0%)	34 (47.9%)	
6–10	34 (24.3%)	17 (24.6%)	17 (23.9%)	
>10	31 (22.1%)	22 (31.9%)	9 (12.7%)	
Change of uniform				0.19
Daily	111 (79.3%)	59 (85.5%)	52 (73.2%)	
Every 2 days	24 (17%)	8 (11.6%)	16 (22.5%)	
More than every 2 days	5 (3.6%)	2 (2.9%)	3 (4.2%)	
Laundering practices				<0.01
Home	132(94.3%)	61 (88.4%)	71 (100.0%)	
Work	8(5.7%)	8 (11.6%)	0 (0.0%)	
Staff movement during shift				0.03
Visit of multiple sites	112 (80%)	50 (72.5)	62 (87.3)	
Toilet use	21 (15%)	12 (17.4)	9 (12.7)	
Visit other wards	6 (4.3%)	6 (8.7)	0 (0.0)	
Visit to the cafeteria	1 (0.7%)	1 (1.4)	0 (0.0)	
Hand washing frequency				0.51
>5 times per shift	137 (97%.9)	68 (98.6)	69 (97.2)	
1–5 times per shift	3 (2.1%)	1 (1.4)	2 (2.8)	

HCW: healthcare workers; LTCF: long-term care facility.

**Table 2:** Isolation of MDRB from HCW in hospitals (*n* = 69) in comparison to LTCF (*n* = 71).

MDRB	Hospital *n* (%)	LTCF *n* (%)	*p*-Value
VRE	3 (4.3)	13 (18.3)	0.01
MRSA	7 (10.1)	8 (14.1)	0.61
ESBL-producing Enterobacterales	0 (0.0)	5 (7.0)	0.06
Carbapenem-resistant *Acinetobacter baumanni*	0 (0.0)	1 (1.4)	1.00

ESBL: extended-spectrum b-lactamase; MDRB: multidrug-resistant bacteria; MRSA: methicillin-resistant *Staphylococcus aureus*; LTCF: long-term care facility; VRE: vancomycin-resistant enterococci.

**Table 3:** HCW characteristics and practices in association with MDRB isolation (*n* = 32).

Characteristics/Practices	MDRB *n* (%)	*p*-Value
Health care setting		0.03
Hospital	10/69 (14.5)	
LTCF	22/71 (31.0)	
Type of HCW		0.29
Nurses	29/131 (22.1)	
Cleaning staff	1/5 (20.0)	
Care assistants	2/4 (50.0)	
Years of work		0.45
<1	5/12 (41.7)	
1–5	12/63 (19.0)	
6–10	8/34 (23.5)	
>10	7/31 (22.6)	
Change of uniform		0.95
Daily	26/111 (23.4)	
Every 2 days	5/24 (20.8)	
Less than every 2 days	1/5 (20.0)	
Laundering practices		0.68
Home	31/132 (23.5)	
Work	1/8 (12.5)	
Staff movement during shift		0.32
Visit of multiple sites	23/112 (20.5)	
Toilet use	8/21 (38.1)	
Visit other wards	1/6 (16.7)	
Visit to the cafeteria	0/1 (0.0)	
Hand washing frequency		0.54
1–5 times per shift	1/3 (33.3)	
>5 times per shift	31/137 (22.6)	

HCW: healthcare workers; LTCF: long-term care facility; MDRB: multidrug-resistant bacteria.

## Data Availability

The data presented in this study are available on request from the corresponding author. The data are not publicly available due to privacy issues concerning participating centers and departments.

## Supplementary Materials

The following are available online at https://www.mdpi.com/article/10.3390/antibiotics11010049/s1. Study questionnaire.
