# Supplementary material for: Multidrug-Resistant Bacteria on Healthcare Workers’ Uniforms in Hospitals and Long-Term Care Facilities in Cyprus
Source: Antibiotics (Basel). 2021 Dec 31;11(1):49. doi: 10.3390/antibiotics11010049 (PMC8773169; doi:10.3390/antibiotics11010049)
Supplement: Supplementary file 1 [file antibiotics-11-00049-s001.zip › antibiotics-1509434-supplementary.pdf]

Supplementary Material: Study questionnaire (Greek)

ΣΧΕΣΗ ΜΕΤΑΞΥ ΜΙΚΡΟ-ΟΡΓΑΝΙΣΜΩΝ ΚΑΙ ΥΦΑΣΜΑΤΩΝ ΣΤΟΝ ΤΟΜΕΑ ΤΗΣ ΥΓΕΙΑΣ- ΚΙΝΔΥΝΟΣ ΕΞΑΠΛΩΣΗΣ ΜΟΛΥΝΣΕΩΝ, ΤΡΟΠΟΙ ΠΕΡΙΟΡΙΣΜΟΥ ΤΟΥΣ

Η ΣΥΛΛΟΓΗ ΔΕΙΓΜΑΤΩΝ ΑΠΟΤΕΛΕΙ ΜΕΡΟΣ ΤΗΣ ΕΡΕΥΝΑΣ ΤΗΣ ΔΙΔΑΚΤΟΡΙΚΗΣ ΜΟΥ ΔΙΑΤΡΙΒΗΣ ΓΙΑ ΤΟ ΠΡΟΓΡΑΜΜΑPhD ΣΤΗΝ ΔΗΜΟΣΙΑ ΥΓΕΙΑ ΣΤΟ ΟΠΟΙΟ ΕΙΜΑΙ ΥΠΟΨΗΦΙΑ ΣΤΟ ΕΥΡΩΠΑΙΚΟ ΠΑΝΕΠΙΣΤΗΜΙΟ ΚΥΠΡΟΥ.

ΕΡΕΥΝΗΤΡΙΑ -ΠΑΥΛΙΝΑ ΛΕΝΑ,MSc. Med. Microbiology(pavlinalena@cytanet.com.cy)

Οι δειγματοληψίες θα αρχίσουν τον Απρίλιο του 2019.

ΕΥΧΑΡΙΣΤΟΥΜΕ ΓΙΑ ΤΗΝ ΣΥΜΜΕΤΟΧΗ ΣΑΣ Η ΟΠΟΙΑ ΕΙΝΑΙ ΕΘΕΛΟΝΤΙΚΗ

- ΘΑ ΘΕΛΑΜΕ ΝΑ ΣΑΣ ΔΙΑΒΕΒΑΙΩΣΟΥΜΕ ΟΤΙ Η ΔΙΑΔΙΚΑΣΙΑ ΣΥΛΛΟΓΗΣ ΤΩΝ ΔΕΙΓΜΑΤΩΝ ΕΙΝΑΙ ΑΠΟΛΥΤΩΣ ΑΣΦΑΛΗΣ ΚΑΙ ΜΗ ΕΠΕΜΒΑΤΙΚΗ ΓΙΑ ΤΟΝ ΣΥΜΜΕΤΕΧΟΝΤΑ.

- ΤΑ ΔΕΙΓΜΑΤΑ ΘΑ ΠΑΡΘΟΥΝ ΜΕ ΤΗΝ ΧΡΗΣΗ ΒΑΜΒΑΚΟΦΟΡΩΝ ΣΤΥΛΕΩΝ(SWABS), ΑΠΛΑ ΑΓΓΙΖΟΝΤΑΣ 2 ΣΗΜΕΙΑ ΤΩΝ ΣΤΟΛΩΝ ΣΑΣ(SCRUBS/WHITECOATS).

- ΤΑ ΔΕΙΓΜΑΤΑ ΘΑ ΜΕΤΑΦΕΡΤΟΥΝ ΣΤΟ ΕΡΓΑΣΤΗΡΙΟ ΤΟΥ ΕΥΡΩΠΑΙΚΟΥ ΠΑΝΕΠΙΣΤΗΜΙΟΥ ΟΠΟΥ ΘΑ ΓΙΝΟΥΝ ΟΙ ΚΑΛΛΙΕΡΓΕΙΕΣ ΤΩΝ ΔΕΙΓΜΑΤΩΝ ΓΙΑ ΤΥΧΩΝ ΑΝΕΥΡΕΣΗ ΜΙΚΡΟ-ΟΡΓΑΝΙΣΜΩΝ.

- ΤΑ ΔΕΔΟΜΕΝΑ ΠΟΥ ΘΑ ΣΥΛΛΕΧΘΟΥΝ ΘΑ ΕΙΝΑΙ ΜΟΝΟ ΔΗΜΟΓΡΑΦΙΚΑ, ΧΩΡΙΣ ΝΑ ΜΠΟΡΕΙ ΝΑ ΓΙΝΕΙ ΤΑΥΤΙΣΗ ΑΤΟΜΩΝ.

- ΘΑ ΧΡΕΙΑΣΤΕΙ ΜΟΝΟ ΜΙΑ ΥΠΟΓΡΑΦΗ ΑΠΟΔΟΧΗΣ ΤΗΣ ΣΥΜΜΕΤΟΧΗΣ ΣΑΣ ΣΤΟ ΕΝΤΥΠΟ ΓΕΝΙΚΩΝ ΠΛΗΡΟΦΟΡΙΩΝ.

- ΟΛΑ ΤΑ ΑΠΟΤΕΛΕΣΜΑΤΑ ΠΟΥ ΘΑ ΕΞΑΧΘΟΥΝ ΘΑ ΕΙΝΑΙ ΑΝΟΙΚΤΑ ΓΙΑ ΠΛΗΡΟΦΟΡΗΣΗ ΣΑ ΣΕ ΗΜΕΡΙΔΕΣ ΠΟΥ ΘΑ ΠΡΟΓΡΑΜΜΑΤΙΣΤΟΥΝ.

ΕΡΩΤΗΜΑΤΟΛΟΓΙΟ ΣΥΜΜΕΤΕΧΟΝΤΑ- **Questionnaire for HCW**

ΦΥΛΟ	Α	Θ	
ΠΕΡΙΟΧΗ ΜΟΝΙΜΗΣ ΔΙΑΜΟΝΗΣ			
ΧΡΗΣΗ ΧΕΡΙΟΥ	ΑΡΙΣΤΕΡΟΧΕΙΡΑΣ		ΔΕΞΕΙΟΧΕΙΡΑΣ
ΝΟΣΟΚΟΜΕΙΟ:		ΝΟΣΗΛΕΥΤΗΡΙΟ:	
ΘΑΛΑΜΟΣ			
ΚΑΘΕ ΠΟΣΕΣ ΜΕΡΕΣ ΓΙΝΕΤΑΙ ΑΛΛΑΓΗ ΣΤΟΛΗΣ	1	2	>2
SCRUBS	WHITE COATS		
ΠΛΗΣΙΜΟ ΣΤΟΛΗΣ	ΣΤΗΝ ΕΡΓΑΣΙΑ	ΣΤΟ ΣΠΙΤΙ	
ΠΛΗΣΙΜΟ ΧΕΡΙΩΝ(ΦΟΡΕΣ ΚΑΤΑ ΤΗΝ ΒΑΡΔΙΑ)	1-3	3-5	>5
ΕΚΤΟΣ ΘΑΛΑΜΟΥ ΠΟΥ ΜΠΟΡΕΙ ΝΑ ΠΑΤΕ ΜΕ ΤΗΝ ΣΤΟΛΗ ΚΑΤΑ ΤΗΝ ΔΙΑΡΚΕΙΑ ΤΗΣ ΒΑΡΔΙΑΣ ΣΑΣ	ΤΟΥΑΛΕΤΑ	ΚΑΦΕΤΕΡΙΑ	ΣΕ ΑΛΛΟΥΣ ΘΑΛΑΜΟΥΣ
ΧΡΟΝΙΑ ΥΠΗΡΕΣΙΑΣ			

ΚΩΔΙΚΟΣ ΔΕΙΓΜΑΤΟΣ:ΗΜΕΡΟΜΗΝΙΑ.

ΥΠΟΓΡΑΦΗ ΣΥΓΚΑΤΑΘΕΣΗΣ
